# Supplementary material for: Plasma Phospholipid n-3/n-6 Polyunsaturated Fatty Acids and Desaturase Activities in Relation to Moderate-to-Vigorous Physical Activity through Pregnancy: A Longitudinal Study within the NICHD Fetal Growth Studies
Source: Nutrients. 2020 Nov 19;12(11):3544. doi: 10.3390/nu12113544 (PMC7699189; doi:10.3390/nu12113544)
Supplement: Supplementary file 1 [file nutrients-12-03544-s001.pdf]

## Supplement tables and figures

**Supplement Table 1.** Maternal plasma phospholipid polyunsaturated fatty acids (PUFAs) composition at each study visits, the NICHD Fetal Growth Studies–Singleton Cohort

| | **Visit 0 (8–13 weeks)** | **Visit 1 (16–22 weeks)** | **Visit 2 (24-29 weeks)** | **Visit 4 (34-37 weeks)** |
|---|---|---|---|---|
| **Sum of n-3 PUFAs** (% of total fatty acids) | 5.39 (0.08) | 5.10 (0.08)* | 5.11 (0.10)* | 4.88 (0.09)* |
| 18:3n-3 (ALA) | 0.21 (0.004) | 0.25 (0.0004)* | 0.27 (0.01)* | 0.26 (0.01)* |
| 20:5n-3 (EPA) | 0.34 (0.01) | 0.19 (0.01)* | 0.31 (0.01) | 0.28 (0.01)* |
| 22:5n-3 (DPA) | 0.69 (0.01) | 0.63 (0.01)* | 0.61 (0.01)* | 0.54 (0.01)* |
| 22:6n-3 (DHA) | 4.16 (0.07) | 4.03 (0.08)* | 3.92 (0.09)* | 3.80 (0.08)* |
| **Sum of n-6 PUFAs** (% of total fatty acids) | 37.08 (0.14) | 36.93 (0.13) | 37.03 (0.16) | 36.88 (0.17)* |
| 18:2n-6 (LA) | 20.67 (0.15) | 21.69 (0.15)* | 21.93 (0.19)* | 22.38 (0.18)* |
| 18:3n-6 (GLA) | 0.08 (0.002) | 0.08 (0.002) | 0.07 (0.002) | 0.07 (0.002) |
| 20:2n-6 (EDA) | 0.50 (0.01) | 0.51 (0.01) | 0.51 (0.01) | 0.46 (0.01)* |
| 20:3n-6 (DGLA) | 3.38 (0.05) | 3.40 (0.04) | 3.40 (0.05) | 3.30 (0.06)* |
| 20:4n-6 (AA) | 11.36 (0.11) | 0.42 (0.12)* | 9.98 (0.14)* | 9.55 (0.13)* |
| 22:4n-6 (DTA) | 0.52 (0.01) | 0.30 (0.01)* | 0.53 (0.01) | 0.48 (0.01)* |
| 22:5n-6 (n6-DPA) | 0.56 (0.01) | 0.53 (0.01) | 0.61 (0.02)* | 0.63 (0.02)* |
| **PUFA ratios** | | | | |
| GLA/LA (Δ6-desaturase) | 0.004 (0.0001) | 0.004 (0.0001)* | 0.003 (0.0001) | 0.003 (0.0001)* |
| AA/DGLA (Δ5-desaturase) | 3.61 (0.07) | 3.27 (0.07)* | 3.11 (0.07)* | 3.12 (0.08)* |

- Data are presented as weighted mean (standard errors, SE).
- Sampling weights were applied to all analyses to represent the full NICHD Fetal Growth Studies–Singletons Cohort.
- *P-values < 0.05 when comparing each visit to visit 0 using paired *t*-tests.
- Abbreviations: PUFA, polyunsaturated fatty acids; AA, arachidonic acid; ALA, alpha-linolenic acid; DGLA, dihomo-gamma-linolenic acid; DHA, docosahexaenoic acid; DPA, docosapentaenoic acid; DTA, docosatetraenoic acid; EDA, eicosadienoic acid; EPA, eicosapentaenoic acid; GDM, gestational diabetes mellitus; GLA, gamma-linolenic acid; LA, linoleic acid; PUFA, polyunsaturated fatty acid

**Supplement Table 2.** Associations of maternal moderate-to-vigorous physical activity (MVPA) and plasma phospholipid polyunsaturated fatty acids (PUFAs) at visit 2 and visit 4 among women without GDM, the NICHD Fetal Growth Studies–Singleton Cohort

| | **Visit 2 (24-29 weeks) (N=107)** | | **Visit 4 (34-37 weeks) (N=102)** | |
|---|---|---|---|---|
| | **β (SE)** | ***P*** | **β (SE)** | ***P*** |
| **MVPA as continuous variable (hour per week)** | | | | |
| **Sum of n-3 PUFAs** (% of total fatty acids) | 0.043 (0.053) | 0.98 | 0.034 (0.067) | 0.80 |
| 18:3n-3 (ALA) | -0.006 (0.004) | 0.13 | -0.002 (0.005) | 0.55 |
| 20:5n-3 (EPA) | 0.005 (0.008) | 0.80 | 0.003 (0.008) | 0.48 |
| 22:5n-3 (DPA) | 0.013 (0.008) | 0.51 | 0.010 (0.009) | 0.72 |
| 22:6n-3 (DHA) | 0.030 (0.048) | 0.99 | 0.023 (0.062) | 0.71 |
| **Sum of n-6 PUFAs** (% of total fatty acids) | **-0.159 (0.099)** | **0.003*** | 0.027 (0.126) | 0.61 |
| 18:2n-6 (LA) | -0.291 (0.119) | 0.02 | -0.154 (0.148) | 0.52 |
| 18:3n-6 (GLA) | 0.002 (0.001) | 0.67 | -0.002 (0.002) | 0.11 |
| 20:2n-6 (EDA) | -0.002 (0.004) | 0.22 | -0.006 (0.005) | 0.64 |
| 20:3n-6 (DGLA) | -0.012 (0.029) | 0.21 | 0.009 (0.039) | 0.46 |
| 20:4n-6 (AA) | 0.115 (0.079) | 0.87 | 0.163 (0.094) | 0.55 |
| 22:4n-6 (DTA) | -0.001 (0.008) | 0.60 | 0.002 (0.010) | 0.74 |
| 22:5n-6 (n6-DPA) | 0.030 (0.011) | 0.12 | 0.016 (0.015) | 0.53 |
| **PUFA ratio** | | | | |
| GLA/LA (Δ6-desaturase) | 0.0001 (0.00007) | 0.38 | -0.00008 (0.00009) | 0.90 |
| AA/DGLA (Δ5-desaturase) | 0.033 (0.040) | 0.46 | 0.036 (0.052) | 0.50 |
| **MVPA high vs. low (reference group)** | | | | |
| **Sum of n-3 PUFAs** (% of total fatty acids) | 0.42 (0.25) | 0.09 | 0.07 (0.27) | 0.79 |
| 18:3n-3 (ALA) | -0.01 (0.02) | 0.72 | -0.001 (0.02) | 0.95 |
| 20:5n-3 (EPA) | 0.08 (0.04) | 0.02 | 0.03 (0.03) | 0.30 |
| 22:5n-3 (DPA) | 0.05 (0.04) | 0.16 | 0.004 (0.04) | 0.91 |
| 22:6n-3 (DHA) | 0.30 (0.23) | 0.19 | 0.04 (0.25) | 0.89 |
| **Sum of n-6 PUFAs** (% of total fatty acids) | -0.89 (0.46) | 0.06 | -0.03 (0.51) | 0.95 |
| 18:2n-6 (LA) | -1.28 (0.56) | 0.02 | -0.42 (0.60) | 0.48 |
| 18:3n-6 (GLA) | 0.01 (0.01) | 0.31 | -0.002 (0.01) | 0.80 |

| | | | | |
|---|---|---|---|---|
| 20:2n-6 (EDA) | -0.01 (0.02) | 0.43 | -0.03 (0.02) | 0.15 |
| 20:3n-6 (DGLA) | -0.06 (0.14) | 0.67 | -0.01 (0.16) | 0.93 |
| 20:4n-6 (AA) | 0.35 (0.38) | 0.35 | 0.37 (0.38) | 0.34 |
| 22:4n-6 (DTA) | 0.02 (0.04) | 0.55 | 0.01 (0.04) | 0.72 |
| 22:5n-6 (n6-DPA) | 0.09 (0.05) | 0.09 | 0.05 (0.06) | 0.38 |
| **PUFA ratios** | | | | |
| GLA/LA (Δ6-desaturase) | 0.0005 (0.0003) | 0.12 | 0.0004 (0.0004) | 0.91 |
| AA/DGLA (Δ5-desaturase) | 0.15 (0.19) | 0.42 | 0.15 (0.21) | 0.48 |

- Multivariable linear regression models adjusted for age (years), race, education, marital status, nulliparous, and pre-pregnancy body mass index (BMI; kg/m2), Alternative Health Eating Index (AHEI) score.
- Individual PUFA is measured as % of total fatty acids.
- Women were classified into high MVPA group if they had ≥ 150 minutes of moderate-to-vigorous physical activity (MVPA) per week.
- *P-value is significant after Bonferroni correction.
- Abbreviations: PUFA, polyunsaturated fatty acids; AA, arachidonic acid; ALA, alpha-linolenic acid; DGLA, dihomo-gamma-linolenic acid; DHA, docosahexaenoic acid; DPA, docosapentaenoic acid; DTA, docosatetraenoic acid; EDA, eicosadienoic acid; EPA, eicosapentaenoic acid; GDM, gestational diabetes mellitus; GLA, gamma-linolenic acid; LA, linoleic acid; PUFA, polyunsaturated fatty acid

**Supplement Table 3.** Associations of change in maternal moderate-to-vigorous physical activity (MVPA) levels between visit 0 and visit 4 and plasma phospholipid polyunsaturated fatty acids (PUFAs) at visit 4 among women without GDM, the NICHD Fetal Growth Studies–Singleton Cohort

| | **Low-Low (N=42)** | **High-High (N=16)** | | **High-Low (N=37)** | | **Low-High (N=7)** | |
|---|---|---|---|---|---|---|---|
| | | **β (SE)** | ***P*** | **β (SE)** | ***P*** | **β (SE)** | ***P*** |
| **Sum of n-3 PUFAs (**% of total fatty acids) | Reference | 0.21 (0.39) | 0.59 | 0.20 (0.25) | 0.43 | -0.16 (0.48) | 0.74 |
| 18:3n-3 (ALA) | Reference | 0.02 (0.03) | 0.55 | -0.003 (0.02) | 0.87 | -0.05 (0.03) | 0.10 |
| 20:5n-3 (EPA) | Reference | 0.02 (0.04) | 0.61 | -0.05 (0.02) | 0.04 | -0.04 (0.04) | 0.28 |
| 22:5n-3 (DPA) | Reference | 0.10 (0.05) | 0.03 | 0.02 (0.03) | 0.47 | -0.06 (0.06) | 0.26 |
| 22:6n-3 (DHA) | Reference | 0.07 (0.35) | 0.84 | 0.23 (0.23) | 0.32 | -0.004 (0.43) | 0.99 |
| **Sum of n-6 PUFAs (**% of total fatty acids) | Reference | -0.83 (0.65) | 0.20 | -0.66 (0.45) | 0.14 | 1.31 (0.71) | 0.06 |
| 18:2n-6 (LA) | Reference | -1.57 (0.72) | 0.03 | -0.94 (0.54) | 0.08 | 0.90 (0.87) | 0.30 |
| 18:3n-6 (GLA) | Reference | 0.003 (0.01) | 0.78 | -0.01 (0.01) | 0.28 | -0.02 (0.01) | 0.04 |
| 20:2n-6 (EDA) | Reference | -0.004 (0.03) | 0.88 | 0.02 (0.02) | 0.38 | -0.07 (0.04) | 0.06 |
| 20:3n-6 (DGLA) | Reference | 0.27 (0.23) | 0.24 | 0.08 (0.14) | 0.57 | -0.48 (0.29) | 0.09 |
| 20:4n-6 (AA) | Reference | 0.38 (0.53) | 0.47 | 0.25 (0.33) | 0.45 | 1.09 (0.58) | 0.06 |
| 22:4n-6 (DTA) | Reference | -0.04 (0.05) | 0.45 | -0.08 (0.03) | 0.01 | -0.02 (0.07) | 0.81 |
| 22:5n-6 (n6-DPA) | Reference | 0.13 (0.09) | 0.17 | 0.03 (0.05) | 0.59 | -0.10 (0.10) | 0.33 |
| **PUFA ratios** | | | | | | | |
| GLA/LA (Δ6-desaturase) | Reference | 0.0004 (0.0005) | 0.45 | -0.0002 (0.0003) | 0.59 | -0.001 (0.0005) | 0.03 |
| AA/DGLA (Δ5-desaturase) | Reference | -0.16 (0.31) | 0.60 | -0.12 (0.19) | 0.55 | 0.85 (0.38) | 0.03 |

- Multivariable linear regression models adjusted for age (years), race, education, marital status, nulliparous, and pre-pregnancy body mass index (BMI; kg/m2), and Alternative Health Eating Index (AHEI) score.
- Individual PUFA is measured as % of total fatty acids.
- *P-value is significant after Bonferroni correction.
- Abbreviations: **PUFA, p**olyunsaturated fatty acids; AA, arachidonic acid; ALA, alpha-linolenic acid; DGLA, dihomo-gamma-linolenic acid; DHA, docosahexaenoic acid; DPA, docosapentaenoic acid; DTA, docosatetraenoic acid; EDA, eicosadienoic acid; EPA, eicosapentaenoic acid; GDM, gestational diabetes mellitus; GLA, gamma-linolenic acid; LA, linoleic acid; PUFA, polyunsaturated fatty acid.

**Supplement Table 4.** Time-specific associations of maternal moderate-to-vigorous physical activity (MVPA) and plasma phospholipid polyunsaturated fatty acids (PUFAs) at each study visits, the NICHD Fetal Growth Studies–Singleton Cohort

| | Visit 0[§] (8-13 weeks) (N=318) | | Visit 1[‡] (16-22 weeks) (N=308) | | Visit 2[‡] (24-29 weeks) (N=207) | | Visit 4[‡] (34-37 weeks) (N=190) | |
|---|---|---|---|---|---|---|---|---|
| | **β (SE)** | ***P*** | **β (SE)** | ***P*** | **β (SE)** | ***P*** | **β (SE)** | ***P*** |
| **MVPA as continuous variable (hour per week)** | | | | | | | | |
| **Sum of n-3 PUFAs** (% of total fatty acids) | 0.008 (0.027) | 0.78 | -0.050 (0.038) | 0.19 | 0.004 (0.038) | 0.92 | 0.001 (0.042) | 0.99 |
| 18:3n-3 (ALA) | -0.0001 (0.001) | 0.95 | 0.001 (0.002) | 0.51 | -0.005 (0.003) | 0.05 | 0.001 (0.003) | 0.86 |
| 20:5n-3 (EPA) | 0.006 (0.004) | 0.11 | 0.002 (0.003) | 0.42 | 0.001 (0.005) | 0.80 | -0.003 (0.005) | 0.55 |
| 22:5n-3 (DPA) | 0.002 (0.005) | 0.68 | -0.011 (0.006) | 0.04 | 0.006 (0.006) | 0.30 | 0.013 (0.005) | 0.02 |
| 22:6n-3 (DHA) | 0.00001 (0.026) | 1.00 | -0.042 (0.035) | 0.22 | 0.002 (0.034) | 0.95 | -0.010 (0.038) | 0.80 |
| **Sum of n-6 PUFAs** (% of total fatty acids) | -0.145 (0.058) | 0.01 | 0.009 (0.061) | 0.88 | **-0.278 (0.068)** | **<0.001*** | -0.092 (0.080) | 0.25 |
| 18:2n-6 (LA) | -0.107 (0.061) | 0.08 | -0.071 (0.074) | 0.34 | **-0.280 (0.081)** | **0.001*** | -0.180 (0.094) | 0.05 |
| 18:3n-6 (GLA) | -0.0001 (0.001) | 0.92 | 0.002 (0.001) | 0.04 | 0.001 (0.001) | 0.56 | **-0.004 (0.001)** | **<0.001*** |
| 20:2n-6 (EDA) | 0.0002 (0.002) | 0.90 | -0.0002 (0.003) | 0.95 | -0.004 (0.002) | 0.15 | -0.001 (0.003) | 0.80 |
| 20:3n-6 (DGLA) | 0.010 (0.019) | 0.59 | 0.014 (0.021) | 0.52 | -0.030 (0.020) | 0.15 | 0.026 (0.027) | 0.34 |
| 20:4n-6 (AA) | -0.043 (0.042) | 0.31 | 0.050 (0.054) | 0.35 | 0.020 (0.058) | 0.73 | 0.053 (0.064) | 0.41 |
| 22:4n-6 (DTA) | -0.003 (0.004) | 0.45 | 0.002 (0.003) | 0.59 | -0.005 (0.006) | 0.42 | 0.001 (0.006) | 0.86 |
| 22:5n-6 (n6-DPA) | -0.002 (0.005) | 0.65 | 0.012 (0.007) | 0.07 | 0.019 (0.007) | 0.01 | 0.012 (0.010) | 0.21 |
| **PUFA ratios** | | | | | | | | |
| GLA/LA (Δ6-desaturase) | 0.00002 (0.00004) | 0.58 | 0.0001 (0.00004) | 0.03 | 0.0001 (0.0001) | 0.15 | -0.0002 (0.0001) | 0.01 |
| AA/DGLA (Δ5-desaturase) | -0.025 (0.026) | 0.35 | -0.008 (0.029) | 0.78 | 0.028 (0.028) | 0.33 | -0.023 (0.039) | 0.55 |
| **MVPA high vs. low (reference group)** | | | | | | | | |
| **Sum of n-3 PUFAs** (% of total fatty acids) | -0.28 (0.14) | 0.06 | -0.32 (0.17) | 0.06 | 0.25 (0.18) | 0.17 | -0.08 (0.19) | 0.69 |
| 18:3n-3 (ALA) | -0.003 (0.01) | 0.65 | 0.01 (0.01) | 0.40 | -0.01 (0.01) | 0.53 | 0.01 (0.01) | 0.32 |
| 20:5n-3 (EPA) | -0.03 (0.02) | 0.12 | -0.002 (0.01) | 0.90 | 0.06 (0.02) | 0.02 | 0.02 (0.02) | 0.42 |
| 22:5n-3 (DPA) | -0.03 (0.02) | 0.22 | -0.06 (0.03) | 0.01 | 0.05 (0.03) | 0.04 | 0.02 (0.02) | 0.33 |
| 22:6n-3 (DHA) | -0.21 (0.13) | 0.12 | -0.26 (0.16) | 0.09 | 0.15 (0.16) | 0.36 | -0.13 (0.18) | 0.45 |

| | | | | | | | | |
|---|---|---|---|---|---|---|---|---|
| **Sum of n-6 PUFAs** (% of total fatty acids) | -0.43 (0.31) | 0.17 | 0.09 (0.27) | 0.73 | **-1.37 (0.33)** | **<0.001*** | -0.46 (0.37) | 0.21 |
| 18:2n-6 (LA) | -0.34 (0.32) | 0.29 | -0.10 (0.34) | 0.76 | **-1.53 (0.39)** | **<0.001*** | -0.44 (0.43) | 0.30 |
| 18:3n-6 (GLA) | -0.002 (0.004) | 0.52 | 0.01 (0.004) | 0.05 | 0.01 (0.004) | 0.05 | -0.01 (0.01) | 0.12 |
| 20:2n-6 (EDA) | -0.00003 (0.01) | 1.00 | -0.0004 (0.01) | 0.97 | -0.03 (0.01) | 0.02 | -0.01 (0.01) | 0.29 |
| 20:3n-6 (DGLA) | 0.06 (0.10) | 0.51 | 0.14 (0.09) | 0.14 | -0.13 (0.10) | 0.18 | 0.03 (0.13) | 0.79 |
| 20:4n-6 (AA) | -0.001 (0.001) | 0.31 | -0.02 (0.24) | 0.93 | 0.23 (0.28) | 0.42 | -0.08 (0.29) | 0.79 |
| 22:4n-6 (DTA) | -0.03 (0.02) | 0.14 | 0.0004 (0.02) | 0.98 | 0.02 (0.03) | 0.60 | 0.01 (0.03) | 0.65 |
| 22:5n-6 (n6-DPA) | 0.05 (0.02) | 0.07 | 0.07 (0.03) | 0.02 | 0.07 (0.03) | 0.04 | 0.04 (0.04) | 0.42 |
| **PUFA ratios** | | | | | | | | |
| GLA/LA (Δ6-desaturase) | -0.0001 (0.0002) | 0.75 | 0.0004 (0.0002) | 0.05 | 0.001 (0.0002) | 0.004 | -0.0003 (0.0003) | 0.34 |
| AA/DGLA (Δ5-desaturase) | -0.18 (0.14) | 0.20 | -0.17 (0.13) | 0.20 | 0.21 (0.14) | 0.13 | -0.04 (0.18) | 0.80 |

- Multivariable linear regression models adjusted for age (years), race, education, marital status, nulliparous, pre-pregnancy body mass index (BMI; kg/m2), Alternative Health Eating Index (AHEI) score, and **time between last time of food intake and specimen acquisition (hours)**±.
- Individual PUFA is measured as % of total fatty acids.
- Sampling weights were applied to all analyses to represent the full NICHD Fetal Growth Studies–Singletons population.
- *P-value is significant after Bonferroni correction.
- § Physical activity in the previous year.
- ǂ Physical activity since the last visit.
- ± Missing values in AHEI were imputed by mean of each visit.
- Abbreviations: PUFA, polyunsaturated fatty acids; AA, arachidonic acid; ALA, alpha-linolenic acid; DGLA, dihomo-gamma-linolenic acid; DHA, docosahexaenoic acid; DPA, docosapentaenoic acid; DTA, docosatetraenoic acid; EDA, eicosadienoic acid; EPA, eicosapentaenoic acid; GDM, gestational diabetes mellitus; GLA, gamma-linolenic acid; LA, linoleic acid; PUFA, polyunsaturated fatty acid
